# Transport Pathways That Contribute to the Cellular Distribution of Phosphatidylserine

**DOI:** 10.3389/fcell.2021.737907

**Published:** 2021-09-01

**Authors:** Guillaume Lenoir, Juan Martín D’Ambrosio, Thibaud Dieudonné, Alenka Čopič

**Affiliations:** ^1^Université Paris-Saclay, CEA, CNRS, Institute for Integrative Biology of the Cell, Gif-sur-Yvette, France; ^2^Centre de Recherche en Biologie Cellulaire de Montpellier (CRBM), Université de Montpellier, CNRS, Montpellier, France; ^3^Danish Research Institute of Translational Neuroscience – DANDRITE, Nordic EMBL Partnership for Molecular Medicine, Department of Molecular Biology and Genetics, Aarhus University, Aarhus, Denmark

**Keywords:** phosphatidylserine, membrane asymmetry, lipid transfer protein, flippase, lipid scramblase, membrane contact site, lipid domain, budding yeast

## Abstract

Phosphatidylserine (PS) is a negatively charged phospholipid that displays a highly uneven distribution within cellular membranes, essential for establishment of cell polarity and other processes. In this review, we discuss how combined action of PS biosynthesis enzymes in the endoplasmic reticulum (ER), lipid transfer proteins (LTPs) acting within membrane contact sites (MCS) between the ER and other compartments, and lipid flippases and scramblases that mediate PS flip-flop between membrane leaflets controls the cellular distribution of PS. Enrichment of PS in specific compartments, in particular in the cytosolic leaflet of the plasma membrane (PM), requires input of energy, which can be supplied in the form of ATP or by phosphoinositides. Conversely, coupling between PS synthesis or degradation, PS flip-flop and PS transfer may enable PS transfer by passive flow. Such scenario is best documented by recent work on the formation of autophagosomes. The existence of lateral PS nanodomains, which is well-documented in the case of the PM and postulated for other compartments, can change the steepness or direction of PS gradients between compartments. Improvements in cellular imaging of lipids and membranes, lipidomic analysis of complex cellular samples, reconstitution of cellular lipid transport reactions and high-resolution structural data have greatly increased our understanding of cellular PS homeostasis. Our review also highlights how budding yeast has been instrumental for our understanding of the organization and transport of PS in cells.

## Introduction

Membranes of eukaryotic cells are composed of numerous lipid species. Many lipids are not homogenously distributed within the cells, but instead are enriched in specific compartments or even in sub-regions of a particular membrane compartment. This is particularly true for phosphatidylserine (PS), a glycerophospholipid with a negatively charged headgroup. Although PS, like many other lipids, is synthesized in the endoplasmic reticulum (ER), it is highly enriched in the plasma membrane (PM) and in late endocytic compartments, in particular in their cytosolic leaflet. Indeed, PS is by far the most abundant anionic phospholipid in the PM and accounts for ∼20–30 mol% of its inner leaflet. PS is thus a key player in the establishment of the “electrostatics” membrane territory in the late secretory pathway (Bigay and Antonny, 2013; Holthuis and Menon, 2014), as opposed to the territory of loose lipid packing and low charge at the ER and *cis*-Golgi. In the “electrostatics” territory, where bilayers are thicker and membrane packing defects are reduced – due to a higher concentration of sterols and saturated sphingolipids – PS fine-tunes interaction of peripheral proteins with membranes. For example, K-Ras, a small GTP binding protein that activates mitogen-activated protein kinase (MAPK) signaling cascade via the effector Raf, is targeted to the PM of mammalian cells via a C-terminal polybasic domain followed by a farnesyl lipid anchor. Ras nanocluster formation is perturbed by depletion of PS from the PM or by PM depolarization (Zhou et al., 2015), resulting in modulation of the signaling output. Similarly, PS controls the nanoclustering of the yeast small GTPase Cdc42, which is essential for the establishment of cell polarity (Sartorel et al., 2018; Meca et al., 2019), and the Rho of Plants (ROP) family member ROP6 (Platre et al., 2019).

In this review, we will discuss cellular mechanisms that contribute to the establishment of PS gradients between and within membrane compartments, primarily focusing on the budding yeast *Saccharomyces cerevisiae*. We will discuss the contribution of PS synthesis and degradation, of the transfer of PS between compartments within membrane contact sites (MCS), and of PS flip-flop between membrane leaflets. We will also review work on the presence of lateral domains of PS within compartments, which introduce additional gradients. Finally, we will highlight some recent research on PS transport during autophagy, which illustrates how different lipid transport pathways combine to distribute PS between membrane compartments.

## Cellular Distribution of PS at Steady State

Early studies of PS subcellular distribution relied on membrane fractionation followed by determination of the lipid content using amine-reactive chemicals in association with biophysical or chromatographic methods [reviewed in Leventis and Grinstein (2010)]. In yeast, a complete study of the lipid composition of subcellular membrane fractions by thin layer chromatography revealed that PS mostly accumulated in the PM (33%) and secretory vesicles (13%), whereas levels in the vacuole, the nucleus, in mitochondria and in microsomes (ER fraction) were around 3–6% (Zinser et al., 1991). Subsequent analysis by mass spectrometry also revealed differences in acyl chain composition of PS in different compartments in yeast, with the PM mostly containing mono-unsaturated PS composed of one oleic (C18:1) and one palmitic acid (C16:0), whereas di-unsaturated PS was most prominent in the nuclear/ER and Golgi membranes (Schneiter et al., 1999). The distribution of PS is similar in mammalian cells, with PS representing ∼10–15 mol% of the total lipid content of the PM (Leventis and Grinstein, 2010). Early biochemical studies using enzymatic degradation by phospholipases, first performed on the PM of red blood cells, also suggested that PS, as well as phosphatidylethanolamine (PE), were almost exclusively located in the cytoplasmic leaflet of this membrane, introducing the concept of transbilayer lipid asymmetry, i.e., a difference in lipid concentration between two leaflets of the same membrane (Verkleij et al., 1973).

Whereas fractionation methods suffer from possible cross-contamination between membranes, higher PS concentration in the PM and the endosomal system of eukaryotic cells was subsequently confirmed in living cells by the use of genetically encoded PS-specific probes, namely the Ca^2+^-independent C2 domain of lactadherin (Yeung et al., 2008; Fairn et al., 2011b) or the PH domain of evectin-2 (Uchida et al., 2011). Comparison of PS surface staining using extracellular fluorescent probes confirmed the largely cytosolic orientation of PS at the PM of mammalian cells at resting state, whereas PS became exposed in the external leaflet in cells undergoing apoptosis (Fadok et al., 1992). A recent study from the Levental group using phospholipase digestion of red blood cells coupled with quantitative mass spectrometry confirmed the strongly asymmetric distribution of PS at the PM, with ∼95% of PS residing in the cytosolic leaflet. This work further revealed a striking difference in acyl chain saturation between the two leaflets, with the outer leaflet containing 35 mol% of saturated lipids, whereas the majority of cytosolic leaflet lipids, including PS, contained poly-unsaturated acyl chains (Lorent et al., 2020), consistent with the fact that PS is highly unsaturated in mammalian cells (Takamori et al., 2006). Such acyl chain asymmetry has been shown to facilitate PM deformation *in silico* (Tiberti et al., 2020).

The transbilayer distribution of PS in endomembranes is less clear, and is particularly controversial for the ER. Because the ER is a biogenic membrane that needs to be able to quickly expand, it was proposed that phospholipids synthesized on the cytoplasmic face of the ER should be rapidly flipped toward the luminal leaflet by an as yet unidentified non-specific and constitutive scramblase, which would be consistent with a symmetrical distribution of phospholipids in the ER membrane (Sanyal and Menon, 2009). However, biochemical studies using microsomal purification, phospholipase treatment and thin layer chromatography found accumulation of PS in the luminal leaflet of the ER in rat liver cells (Higgins and Dawson, 1977; Bollen and Higgins, 1980). More recently, electron microscopy has been used to address this issue. Using an on-section approach and purified Lact-C2 protein fused to GST as an epitope for immunogold labeling, Fairn et al. (2011b), in agreement with the earlier biochemical studies, showed accumulation of PS in the luminal side of the ER and the Golgi apparatus, whereas PS became exposed cytosolically at the trans-Golgi network.

Freeze-fracture replica labeling (FRL) methods have also been developed. Quick freezing of the specimen minimizes membrane reorganization during sample preparation. The membrane is then split into two leaflets, lipids and proteins are fixed on a metal cast and lipid distribution is examined by electron microscopy using specific lipid probes that are recognized by gold-conjugated antibodies (Fujimoto et al., 1996; Fujita et al., 2010). Such approach indicated that in human red blood cells, phosphatidylcholine (PC) and sphingomyelin were exclusively located in the outer PM leaflet, whereas PE, PS, phosphatidylinositol (PI), and phosphatidylinositol 4,5-bisphosphate (PI(4,5)P_2_) were only found in the cytosolic leaflet (Murate et al., 2015), in agreement with previous studies using phospholipases. PS was also detected almost exclusively in the cytosolic leaflet of the PM in human skin fibroblasts (Murate et al., 2015; Tsuji et al., 2019). However, Tsuji et al. (2019) found PS labeling predominantly in the cytoplasmic leaflet of the ER in mouse embryonic fibroblasts, unless the cells were treated with a Ca^2+^ ionophore, contradicting the results of Fairn et al. (2011b). In yeast, using the evectin-2 PH probe they found PS more or less evenly distributed between the two leaflets of the ER, the nuclear membrane and the mitochondria. In contrast, the labeling of the cytoplasmic leaflet of the Golgi and the vacuole was much more pronounced than the luminal leaflet, indicative of a strong asymmetric distribution of PS in those organelles. In the PM and the vacuolar membranes, only cytosolic leaflets showed PS accumulation (Tsuji et al., 2019).

The group of Fujimoto further delved into transbilayer lipid asymmetry and its role in autophagosome formation (Orii et al., 2021). They evaluated PS, PC, and phosphatidylinositol 4-phosphate (PI(4)P) asymmetry in yeast autophagosomal membranes, autophagic bodies and vacuoles. They confirmed that PS is largely confined to the cytosolic leaflet of the vacuole in normal growth conditions but, interestingly, in conditions favoring autophagy, PS, PI(4)P, and PC were found evenly distributed over the two leaflets of autophagosomes and autophagic bodies, suggesting that a general mechanism enables transbilayer phospholipid movement in isolation membranes/autophagosomes. This question will be further discussed in Section “Lateral Organization of PS in Membranes.”

Altogether, these various studies indicate that, in healthy cells (i.e., not undergoing apoptosis), PS accumulates in the cytoplasmic leaflet of the PM. However, the exact content and distribution of PS in endomembranes is less clear. Further studies will be needed to resolve these discrepancies, as well as a better understanding of the mechanisms that govern PS homeostasis.

## Ps Synthesis and Degradation

**FIGURE 1:**
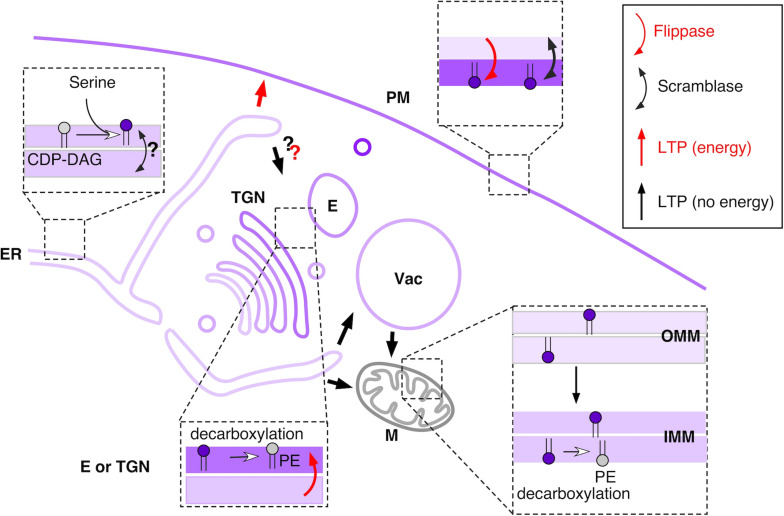
Overview of main PS metabolic and transport pathways in budding yeast. See text for details. Shades of purple indicate approximate relative PS concentration in different membranes or leaflets. PS is represented with a purple headgroup. Red arrows indicate PS transport pathways that require energy (transport against concentration gradient), whereas black arrow indicate passive PS transport. E – endosome, IMM – inner mitochondrial membrane, M – mitochondria, OMM – outer mitochondrial membrane, TGN – *trans*-Golgi network, Vac – vacuole.

Many pathways contribute to the cellular metabolism of PS, with parallels but also differences between yeast and mammalian cells (Acoba et al., 2020; Vance, 2020). In yeast, PS synthesis proceeds *via* a single pathway, where the rate-limiting step of transfer of phosphatidyl group from CDP-diacyglycerol to L-serine is catalyzed by the integral ER membrane PS synthase Cho1 (Henry et al., 2012) (Figure 1). Interestingly, this enzyme is not essential: although the growth of *cho1Δ* cells is severely affected and the cells display defects in cell polarity, yeast cells can survive without PS (Hikiji et al., 1988; Fairn et al., 2011a). Similar observations have been made in plants (Platre et al., 2018).

Several lines of evidence suggest that the active site of Cho1 faces the cytosol. First, both substrates for this reaction are synthesized in the cytosolic environment, CDP-diacylglycerol in the cytosolic leaflet of the ER by Cds1 (Tamura et al., 2013) and L-serine in the cytosol by dephosphorylation of 3-phosphoserine by Ser2 (Albers et al., 2003). Second, Cho1 belongs to the CDP-alcohol phosphatidyltransferase (CAPT) superfamily (pfam01066) of enzymes, which share the 25-30 amino acid long CAPT motif in their active site. Topology predictions place the CAPT motif of Cho1 in the cytosolic side of the ER (Bochud and Conzelmann, 2015), in agreement with analyses of phosphorylation and ubiquitylation sites (Swaney et al., 2013). Interestingly, whereas Cho1-GFP fusion localized throughout the ER, Cho1 enzymatic activity was shown to be significantly increased in PM-associated and mitochondria-associated ER membranes (PAM and MAM), suggesting localized synthesis of PS (Vance, 1990; Gaigg et al., 1995; Pichler et al., 2001).

In mammals, PS synthesis proceeds via two pathways involving PS synthases-1 and 2 (PSS1 and PSS2), which are both enriched in MAM, and which catalyze the exchange of serine for choline of PC and for ethanolamine of PE, respectively (Leventis and Grinstein, 2010). Point mutations in PSS1 lead to a rare genetic disease, the Lenz–Majewski syndrome, and correlate with increased levels of cellular PS (Sohn et al., 2016). This effect is explained by product inhibition of PSS1 by PS, which is alleviated by the Lenz–Majewski mutations. Interestingly, these mutations are predicted to affect protein regions exposed to the ER lumen, opening topological questions regarding the mechanism of PS synthesis by PSS1. In yeast, Cho1 was also suggested to be negatively regulated by PS (Kannan et al., 2017) as well as by phosphorylation via PKA (Henry et al., 2012).

Cho1 levels are also regulated at the transcriptional level by the Henry regulatory circuit, which involves the repressor Opi1 and the Ino2-Ino4 activator complex (Henry et al., 2012). PA levels in the ER control the circuit by mediating the recruitment of Opi1 and allowing Cho1 expression during exponential phase, whereas in stationary phase, PA is used for synthesis of triacylglycerol and Opi1 represses the expression of lipid biosynthetic genes, including Cho1 (Loewen et al., 2004; Hofbauer et al., 2018).

In both yeasts and mammals, PS metabolism has been linked to the metabolism of phosphoinositides via the PI 4-kinase Stt4 (Trotter et al., 1998) and the PI(4)P phosphatase Sac1, which localizes to the ER. In mammals, increased PS levels in the ER directly activate Sac1 (Sohn et al., 2016), and in yeast, deletion of Sac1 was shown to decrease PS levels and change its cellular distribution (Tani and Kuge, 2014).

In yeast as well as in at least some mammalian cell types, the main pathway of PS consumption is *via* decarboxylation into PE (Vance, 2020) (Figure 1). Mammalian genomes encode a single PS decarboxylase, PISD, which localizes to the inner mitochondrial membrane (Kuge et al., 1991). Deletion of this gene in mice leads to abnormal mitochondrial morphology and embryonic lethality (Steenbergen et al., 2005). Two PS decarboxylases, Psd1 and Psd2, are present in yeast; *psd1Δ psd2Δ* cells are auxotrophic for ethanolamine, because PE can only be generated *via* the Kennedy pathway (Storey et al., 2001). Psd1 has been localized to the inner mitochondrial membrane, where high PE levels, particularly generated at this location, are important for maintaining proper mitochondrial function (Bürgermeister et al., 2004; Calzada et al., 2019). However, a recent study suggests that a fraction of Psd1 resides at the ER, and that the ratio between the two pools is modulated according to metabolic needs (Friedman et al., 2018). Similarly, an alternatively-spliced form of the mammalian PISD has been shown to localize to lipid droplets (Kumar et al., 2021). For the second yeast decarboxylase, Psd2, early reports using subcellular fractionation suggested that it localized to the Golgi and the vacuole (Trotter et al., 1995), which is supported by the presence of a Golgi retention sequence in Psd2. More recent work suggests primarily endosomal localization for Psd2 (Gulshan et al., 2010; Ma et al., 2018; Wang et al., 2020). It is clear that decarboxylation of PS to PE requires export out of the ER to reach Psd1 and Psd2 or PISD in mammalian cells.

PS can also be deacylated into lyso-PS, which can be coupled with acyl chain remodeling, but these reactions are not well explored. Two yeast phospholipases B, Pbl2, and Pbl3, are reported to have broad phospholipid specificity (Henry et al., 2012). Another phospholipase B with broad specificity, Lbl1, has been suggested to function on lipid droplets (Selvaraju et al., 2014). In mammalian cells, lyso-PS has been shown to act as a signaling molecule and can be further degraded into glycerol-phosphoserine and a free fatty acid (Omi et al., 2021). Finally, phospholipids can be degraded via the autophagy pathway by the lipase Atg15 residing in the vacuole, but the function of this enzyme also remains to be explored (Hirata et al., 2021).

## Export of PS From the ER

The overall low concentration of PS at the ER suggests that the majority of PS is exported out of the ER after synthesis. One major way of lipid transport is via the vesicular pathway; however, vesicles in general lack mechanisms for selective transport of lipids, therefore vesicular transport will tend to equilibrate lipid composition of different compartments. In contrast, lipid transfer proteins (LTPs) can be highly selective for specific lipid species and can create strong lipid gradients, notably by coupling the transport of two or more lipid species (Wong et al., 2019; Lipp et al., 2020). Whereas vesicles will maintain the bilayer distribution of a phospholipid, LTPs only have access to one bilayer leaflet, i.e., the cytosolic leaflet in the case of cytosolic LTPs. By selectively depleting PS from the cytosolic leaflet of the ER or other compartments, and enriching it in cytosolic leaflets of other compartments, LTPs will therefore influence the transbilayer asymmetry.

Lipid transfer proteins usually function within MCS, regions of close apposition between two (or sometimes more) compartments, which are stabilized by protein tethers that simultaneously contact two compartments (Wong et al., 2017; Wu et al., 2018). Because the ER forms MCS with most other cellular compartments, PS can potentially be directly transferred anywhere in the cell via an LTP (Figure 1).

The first MCS was identified between the ER and the mitochondria. Pioneer work by Jean Vance suggested that the MAM fraction of the ER is involved in lipid exchange between the two compartments (Vance, 1990). Because PS is used as a substrate for synthesis of PE by the PS decarboxylase Psd1 in the inner mitochondrial membrane, LTPs must exist to transfer PS from the ER to the mitochondria. Early biochemical studies identified protein fractions displaying PS-transfer activity (Butler and Thompson, 1975; Lafer et al., 1991). Within the mitochondria, PS transfer from the outer to the inner mitochondrial membrane, mediated by Ups2 and Mdm35, has been well characterized (Watanabe et al., 2015; Aaltonen et al., 2016). In contrast, the identity of LTP(s) that transfer PS from the ER to the mitochondria remains highly debated (see Acoba et al., 2020, for a recent in-depth review).

**FIGURE 2:**
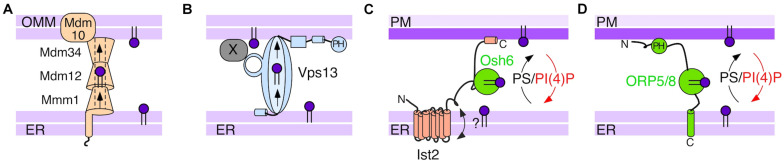
Schematic representation of proteins implicated in PS transfer in the cytosol. **(A)** The yeast ERMES complex tethers the ER to the outer mitochondrial membrane (OMM) and could mediate passive flow of PS via a hydrophobic tunnel. This model is adapted from Kawano et al. (2017). **(B)** Vps13 acts as a lipid bridge between the ER and different compartments, which it targets by interacting with compartment-specific adaptors (X). The yeast protein interacts directly with the ER membrane (Dziurdzik and Conibear, 2021). **(C)** Osh6 in cooperation with Ist2 utilizes the PI(4)P gradient to transfer PS. **(D)** ORP5 and ORP8 represent the closest mammalian orthologues of Osh6. They use a PH domain and a transmembrane domain to bind to the PM and the ER, respectively.

In yeast, the ERMES complex, which tethers the ER and the mitochondria, was proposed to mediate transfer of PS and PC via a hydrophobic tunnel formed by the SMP (synaptotagmin-like mitochondrial lipid-binding protein) domains of ERMES subunits Mdm12, Mdm34, and Mmm1 (Kornmann et al., 2009; AhYoung et al., 2015; Jeong et al., 2017; Kawano et al., 2017) (Figure 2A). However, ERMES mutants do not display a strong defect in phospholipid distribution, suggesting that additional proteins must be involved in PS transfer to mitochondria. Furthermore, the ERMES complex is not conserved in metazoans.

A number of studies suggest that an important lipid transfer route between the ER and the mitochondria in yeast proceeds *via* the vacuole and is mediated by vCLAMPs (vacuole and mitochondria patch). The MCS is formed by Vps39, which bridges the interaction between Ypt7 on the vacuolar and TOM40 on the mitochondrial membrane (Elbaz-Alon et al., 2015; Montoro et al., 2018; Acoba et al., 2020). Disruptions of ERMES and vCLAMPs show synergistic effects, suggesting that the two complexes mediate complementary lipid transfer pathways. The large multi-subunit protein Vps13, which has four human orthologs (VPS13A-D), has been shown to function in the same pathway as vCLAMPs, and can be observed at vacuole-mitochondria MCS as well as at the nucleus-vacuole junction (Dziurdzik and Conibear, 2021). Like ERMES, Vps13 can tether two compartments by simultaneously binding to the ER and to the membrane of another compartment (directly or via an adaptor protein) (Figure 2B). Interestingly, Vps13 is also important for the formation of the prospore membrane during sporulation (Park and Neiman, 2012). Biochemical and structural work suggests that the N-terminal part of Vps13 mediates transfer of glycerophospholipids by forming an elongated tube with a hydrophobic groove that can act as a bridge for lipid crossing (Kumar et al., 2018; Li et al., 2020). Such set-up would be very efficient and shared with the autophagy protein Atg2, which is discussed at the end of this review. However, Vps13 does not appear very selective and it has been suggested that it could mediate bulk-flow of lipids to support membrane expansion by coupling its activity with lipid synthesis on the donor and a lipid sink on the acceptor membrane (Lees and Reinisch, 2020). This model would be compatible with observations that PS synthesis is enriched in the MAM fraction (Vance, 1990) and that targeting of a heterologous PS synthase to ER-mitochondria contacts promotes PS transfer to the mitochondria (Kannan et al., 2017). Furthermore, on the mitochondrial side, decarboxylation of PS to PE would provide a sink in the acceptor compartment, provided that PS can be flipped to the inner leaflet of the outer mitochondrial membrane. To what extent Vps13 participates in the specific transfer of PS remains to be determined.

The studies on lipid transfer between the ER and the mitochondria illustrate the complexity and plasticity of lipid transport pathways, where lipids can reach the same compartment via different routes, and one pathway can often compensate for another.

In 2013, the group of Anne-Claude Gavin presented clear evidence that two highly homologous yeast proteins, Osh6 and Osh7, bind PS and mediate its transfer between the ER and the PM, consistent with their enrichment at the cortical ER, which represents ER-PM contact sites (Schulz et al., 2009; Maeda et al., 2013). These two LTPs belong to a family of seven yeast Osh proteins, homologous to the ORP family of oxysterol-binding protein–related proteins. The ORP/Osh proteins, which are present in all eukaryotes, are characterized by a conserved lipid-binding domain (ORD, for oxysterol-binding protein–related domain). Based on the observation that yeast lacking six of the seven Osh proteins remain viable and lack any strong phenotypes, Osh proteins were initially thought to play redundant roles in the maintenance of cellular lipid homeostasis (Beh et al., 2001). However, Osh proteins can be observed in different cellular locations (Olkkonen and Li, 2013), and crystal structures have shown that, despite their overall similar fold, different ORDs can accommodate different lipid species, phospholipids and/or sterols (Im et al., 2005; de Saint-Jean et al., 2011; Maeda et al., 2013; Tong et al., 2013; Moser Von, Filseck et al., 2015; Delfosse et al., 2020). The apparent redundancy between Osh proteins must therefore rather be due to a redundancy in lipid transport pathways and mechanisms that drive lipid enrichment in different membranes, and/or to compensatory effects between different lipid species. However, it also cannot be excluded that, in cells, Osh proteins can be more promiscuous with regards to the lipid species than what has been reported with minimal systems *in vitro*.

Based on the conservation of critical histidine residues in all ORD’s, the common ligand for all Osh/ORP proteins is likely PI(4)P (Raychaudhuri and Prinz, 2010; Delfosse et al., 2020). It was first demonstrated for Osh4 and its mammalian orthologue OSBP that their ORDs can interchangeably accommodate a sterol or a PI(4)P molecule, leading to a model whereby the two lipids are exchanged in a single transfer cycle, with PI(4)P supplying the energy required for transport of the counter lipids against its concentration gradient, i.e., transfer of sterol from the ER to the trans-Golgi network in the case of Osh4 and OSBP (de Saint-Jean et al., 2011; Mesmin et al., 2013). Similarly, Osh6, as well as its closest mammalian orthologues ORP5 and ORP8, bind and transport PI(4)P, mediating counter-exchange with PS (Chung et al., 2015; Moser Von, Filseck et al., 2015) (Figures 2C,D). Because PI(4)P is continuously generated at the PM by the PI-kinase Stt4 and hydrolyzed at the ER by the PI(4)P phosphatase Sac1 (Foti et al., 2001; Zewe et al., 2018), the gradient of PI(4)P between these two compartments allows Osh6 to transfer PS from the PS-poor ER to the PS-rich PM. The same holds for the transfer of sterol by Osh4/OSBP from the ER to the trans-Golgi network. An acidic patch in the amino-terminal region of Osh6, which forms a lid over the lipid-binding pocket, acts as an electrostatic switch, allowing Osh6 to limit its interaction with the negatively charged PM after lipid extraction to execute cycles of PS-PI(4)P exchange and promote build-up of PS at the PM (Lipp et al., 2019).

Phylogenetic analyses suggest that ORP9, 10 and 11 could also bind to PS due to conserved features that they share with Osh6/7 and ORP5/8 (Raychaudhuri and Prinz, 2010; Maeda et al., 2013). In agreement, a recent study implicates ORP10 in PS transfer between the ER and the trans-Golgi network (Venditti et al., 2019).

It is not clear whether Osh6/7 and their mammalian orthologues always utilize PI(4)P as a counter-ligand for transfer of PS; one could imagine that PI(4)P may not be required if PS were to be transported along its concentration gradient. For example, ORP5 and ORP8 were suggested to mediate PS transfer from the ER to mitochondria, where PS is then decarboxylated, assuring continuous clearance of PS (Galmes et al., 2016). The mechanistic details of this proposed pathway remain to be resolved.

A recent study has suggested that Sfh1, a homologue of the PI/PC transfer protein Sec14, mediates the transfer of PS to endosomes, where PS is converted into PE by Psd2, and also the reverse delivery of PE back to the ER (Mizuike et al., 2019). Another protein from the same family, Pdr17/Sfh4, physically interacts with Psd2 and genetic evidence suggests that Sfh4, Psd2, and Osh6/7 function in the same pathway (Wang et al., 2020). Other genetic evidence suggests that PS is supplied to Psd2 via endocytosis from the PM (Costanzo et al., 2016; Wong et al., 2021), and endosomal recycling is important in maintaining a PE/PS equilibrium (Ma et al., 2018). The roles of Sfh1 and Sfh4 in PS transport remain to be resolved.

## Organelle Targeting of PS Transfer Proteins

Localization of LTPs to contacts between two compartments is a straightforward way to control lipid targeting and can also enable coordination of activities of different LTPs (Hanada, 2018; Quon et al., 2018). Another possible benefit is to promote the rate of lipid transfer, although this may be more affected by the rate-limiting step of lipid extraction (Wong et al., 2017).

As in the case of Vps13 and the multi-subunit ERMES complex, several Osh proteins and the majority of ORPs contain additional domains and targeting sequences outside of their ORDs that mediate the targeting to MCS (Figure 2). ORP5 and ORP8 localize to the ER through a transmembrane domain downstream of their ORD and they rely on a pleckstrin homology (PH) domain for interaction with the PM via phosphoinositides (Chung et al., 2015; Sohn et al., 2018) (Figure 2D). PH domains are used by many proteins, including Vps13, Osh1, Osh2, Osh3, and the majority of ORPs, to interact with negatively charged membrane lipids or lipids in combination with the small GTPase Arf1 (Levine and Munro, 2002; Dziurdzik and Conibear, 2021). However, the transmembrane domain of ORP5/8 is an exception among LTPs (Delfosse et al., 2020), because the other multi-domain Osh/ORP proteins interact with the ER using a short “FFAT” motif (two phenylalanine in an acidic tract), which binds to the cytosolic domain of the integral ER protein VAP (Scs2 and Scs22 in yeast) (Loewen et al., 2003). VAP proteins interact with a multitude of LTPs and other proteins and therefore represent a major mechanism for protein localization to the ER (Slee and Levine, 2019). A FFAT motif, or a phosphorylated variant, is used by mammalian VPS13 proteins to contact the ER (Kumar et al., 2018; Guillén-Samander et al., 2021). A putative FFAT motif has also been identified in the yeast Vps13 sequence (Slee and Levine, 2019), and it has also been suggested that the yeast Vps13 could interact directly with the lipid surface of the ER (Dziurdzik and Conibear, 2021).

Some LTPs can target several compartments by interacting with different binding partners. The most striking example is Vps13, which can bind to a number of adaptor proteins residing on different membranes using its adaptor-binding β-propeller domain (Park and Neiman, 2012; John Peter et al., 2017; Bean et al., 2018) (Figure 2B). In mammalian cells, VPS13A and VPS13D were shown to localize to the ER-mitochondria contacts using different mechanisms. The localization of VPS13A requires its C-terminal PH domain (Kumar et al., 2018), whereas VPS13D uses the β-propeller domain to interact with the mitochondrial GTPase Miro (Guillén-Samander et al., 2021). Miro is an orthologue of Gem1, which interacts with ERMES in yeast (Kornmann et al., 2011), suggesting that VPS13D could replace the function of ERMES in mammalian cells. Splice variants of Miro also localize to peroxisomes, implicating VPS13D in phospholipid transfer to peroxisomes, which have been shown to require non-vesicular lipid transfer for their growth (Raychaudhuri and Prinz, 2008). ORP5/8 have been proposed to localize to the MCS between the ER and mitochondria by interacting with the mitochondrial protein PTPIP51 via their ORD (Galmes et al., 2016). A recent study reports ORP5 in the contacts between the ER and the lipid droplets (Du et al., 2019).

Although Osh6 and Osh7 contain only an ORD domain, they show a well-defined localization at the ER-PM contacts (Schulz et al., 2009; Maeda et al., 2013). We have recently demonstrated that the localization of Osh6 to this MCS is mediated by another protein, Ist2 (D’Ambrosio et al., 2020). Importantly, the interaction between Osh6/7 and Ist2 is required for their PS transfer activity in cells (D’Ambrosio et al., 2020) and for the subsequent processing of PS into PE by Psd2 (Wong et al., 2021) (Figure 2C). Unlike the adaptors mentioned earlier, Ist2 is itself a tethering protein between the ER and the PM. Its deletion reduces the amount of ER-PM contact and leads to an increase in cellular PI(4)P levels (Manford et al., 2012; Wolf et al., 2012). The N-terminal domain of Ist2 is embedded in the ER, followed by a disordered cytosolic tail close to 300 amino acids in length, and finally a polybasic region that interacts with the PM (Fischer et al., 2009; Maass et al., 2009; Kralt et al., 2014). Interestingly, disordered domains are common in proteins that populate MCS (Jamecna et al., 2019). Recently, two teams employed high resolution imaging to explore the organization of yeast ER-PM contact sites and found that Ist2 displayed a slight preference for flat ER sheets (Collado et al., 2019; Hoffmann et al., 2019). A short segment of about 30 amino acids in the middle portion of the tail, which is conserved in yeasts, is sufficient to localize Osh6, and small mutations in this segment functionally mimic deletion of Osh6/7 (D’Ambrosio et al., 2020). Total PS levels were substantially decreased in *ist2Δ* and in *osh6Δ osh7Δ* cells, which can be explained by the product inhibition of the PS synthase Cho1 (Kannan et al., 2017) and suggests that this transport pathway removes a large fraction of PS from the ER.

Whereas the cytosolic tail of Ist2 is unique to fungi (D’Ambrosio et al., 2020), the ER-embedded domain, which contains ten predicted transmembrane helices, bears homology to the TMEM16 protein family (Brunner et al., 2014). These proteins have been shown to function as Ca^2+^-activated lipid scramblases and/or ion channels (see Section 5), raising the possibility that the transmembrane domain of Ist2 could function in the regulation of lipid homeostasis at the ER. However, reconstitution of purified Ist2 into proteoliposomes did not reveal any lipid scramblase activity under the experimental conditions used, either in the presence or absence of Ca^2+^ (Malvezzi et al., 2013). It cannot be excluded that Ist2 could be activated in a different manner. Therefore the function and regulation of the transmembrane domain of Ist2 remain to be determined.

## Transbilayer Movement of PS by Flippases and Scramblases

Due to their amphipathic nature, phospholipids including PS show extremely slow spontaneous transbilayer movements (or flip-flop) with half-times ranging from several hours to days depending on the nature of the phospholipid headgroup (Holthuis and Levine, 2005). The polar headgroup has to overcome the high energy barrier posed by the hydrophobic core of the membrane formed by lipid acyl chains. However, many processes, such as membrane expansion mediated by lipid synthesis or lipid transfer, require rapid flip-flop of phospholipids across membranes to overcome the gain or loss of lipids in one leaflet vs. the other one. PS flip-flop has also been shown to be important for the regulation of vesicular membrane traffic (Chen et al., 2010; Uchida et al., 2011; Xu et al., 2013; Hankins et al., 2015).

Eukaryotic cells have evolved three types of membrane transporters that mediate the transbilayer flip-flop of lipids (Montigny et al., 2016). First, scramblases transport lipids in a non-selective, bidirectional and energy-independent manner to equilibrate the composition of the two leaflets. In contrast, floppases and flippases use ATP hydrolysis to actively translocate specific lipids unidirectionally against their concentration gradients, hence creating or maintaining transbilayer lipid asymmetry. Floppases transport lipids from the cytoplasmic to the exoplasmic (external or luminal) leaflet of membranes; flippases transport lipids in the opposite direction, i.e., from the exoplasmic to the cytoplasmic leaflet. We will focus on scramblases and flippases as no floppase has been shown to regulate PS distribution.

Diverse transmembrane proteins have been implicated in lipid scrambling. These include members of the Ca^2+^-regulated TMEM16/anoctamin protein family, which also act as ion channels (Malvezzi et al., 2013, 2018; Brunner et al., 2014; Lee et al., 2018; Bushell et al., 2019; Kalienkova et al., 2019), a few G protein-coupled receptors (GPCR) (Menon et al., 2011; Goren et al., 2014), and, recently, the autophagy protein Atg9 (Maeda et al., 2020; Matoba et al., 2020) and the ER protein complex TMEM41B/VMP1 (Li et al., 2020; Ghanbarpour et al., 2021; Huang et al., 2021). Upon reconstitution in proteoliposomes, all these proteins catalyze lipid scrambling, suggesting that this activity is intrinsic and does not require protein co-factors. The Xk-family protein Xkr8 was shown to facilitate PS exposure in apoptotic cells by a mechanism that involves cleavage by caspases (Suzuki et al., 2013, 2016) or activation via phosphorylation near the caspase recognition site (Sakuragi et al., 2019). Xkr9, a paralog of Xkr8, was also shown to promote lipid scrambling in cultured cells (Suzuki et al., 2014). However, a recent study (Straub et al., 2021) has failed to demonstrate lipid flip-flop activity of purified and reconstituted Xkr9, and the demonstration that Xkr8 is a bona fide lipid scramblase is also still awaiting reconstitution in synthetic vesicles.

**FIGURE 3:**
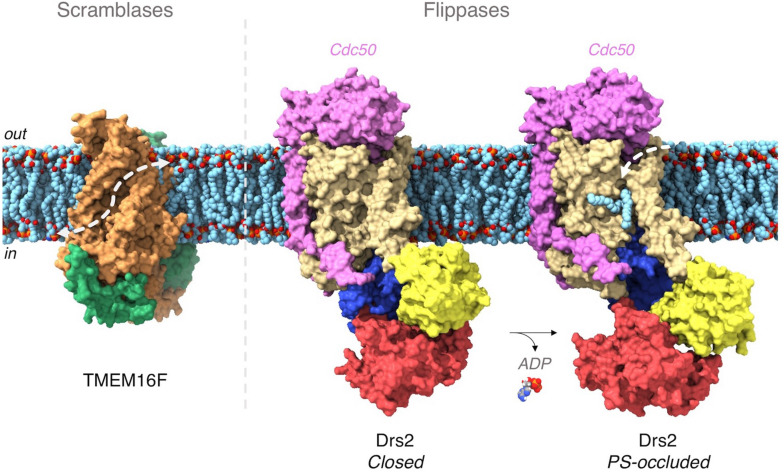
Lipid transport pathway in TMEM16 scramblases and P4-ATPase flippases. In TMEM16 proteins, each monomer (in green and orange, respectively) harbors a cavity (underscored by the dashed white arrow) lined by hydrophilic residues and forming a groove through which lipid headgroups can transit from one leaflet to the other – known as the credit-card model (Pomorski and Menon, 2006). In P4-ATPases, ATP binds to the nucleotide binding domain (in red). The bound ATP molecule then phosphorylates a conserved aspartate in the phosphorylation domain (in blue). Phosphorylation triggers a rotation of the actuator domain (in yellow) which leads to the opening of a lipid hemichannel toward the exoplasmic side of the membrane. The Cdc50 subunit of Drs2 is in pink. (PDB codes used for this figure, nhTMEM16F: 4WIT, Drs2/Cdc50: 7OH7 (closed), 7OH6 (PS-occluded). The figure was generated with ChimeraX (Pettersen et al., 2021).

Among scramblases, members of the TMEM16 family are by far the best characterized (Kalienkova et al., 2021). In 2014, the first crystal structure of nhTMEM16 from *Nectria haematococca* revealed a protein dimer featuring a hydrophilic groove on the edge of each subunit. As this groove connects both leaflets, it suggested a path along which the lipid polar headgroups might slide along (Figure 3) (Brunner et al., 2014). This hypothesis has been reinforced by biochemical studies and molecular dynamics simulations (Bethel and Grabe, 2016; Lee et al., 2018). In addition, recent cryo-EM structures explain the mechanism by which Ca^2+^ binding stimulates lipid transport and also show that the surrounding membrane is deformed by the protein. Thinning of the membrane in the vicinity of the protein is associated with defects in lipid packing, suggesting additional means for lowering the energetic barrier for lipid translocation (Alvadia et al., 2019; Bushell et al., 2019; Falzone et al., 2019; Feng et al., 2019; Kalienkova et al., 2019).

Whereas most TMEM16 proteins localize to the PM, two members of the family have been suggested to function at the ER or in MCS between the ER and other compartments. TMEM16K has been shown to localize predominantly to the ER. As aforementioned, Tsuji et al. observed equilibration of PS levels within the two ER leaflets when wild-type cells were treated with the Ca^2+^ ionophore A23187, but not when A23187 was added to TMEM16K−/− cells (Tsuji et al., 2019), suggesting that TMEM16K is a Ca^2+^-regulated ER scramblase. Further reconstitution into chemically-defined liposomes confirmed that ability of TMEM16K to scramble lipids and the stimulatory effect of Ca^2+^. PC and PE labeled with a fluorescent NBD (nitrobenzoxadiazole) moiety are transported equally well whereas the rate of NBD-PS transport is somewhat slower (Bushell et al., 2019). Another recent study suggests that TMEM16K functions in the contacts between the ER and endosomes and is important for regulation of endosomal traffic (Petkovic et al., 2020). A related protein, TMEM16H/ANO8 has been suggested to function as an ER-PM tether to regulate Ca^2+^ signaling, but the molecular details of this tethering activity remain to be resolved (Jha et al., 2019). Based on these studies, TMEM16K appears as a strong candidate for the long sought-after ER scramblase activity. Recent evidence indicates that the integral membrane protein complex TMEM41B/VMP1 also acts as an ER scramblase (Li et al., 2020; Ghanbarpour et al., 2021; Huang et al., 2021), suggesting that both proteins could sustain the biogenic function of the ER.

Beyond the ER, enrichment of PS in the cytosolic membrane leaflet of healthy cells is catalyzed by the P4 subtype of the ubiquitous P-type ATPase family (P4-ATPases). Interestingly, P-type ATPases were initially identified as cation transporters, but have recently been recognized as lipid transporters (Tang et al., 1996; Coleman et al., 2009; Zhou and Graham, 2009; Dyla et al., 2020). Most P4-ATPases form heterodimers with proteins from the Cdc50 family, which help in targeting the mature complex to its correct subcellular localization.

Recent cryo-EM and X-ray high-resolution structures of P4-ATPases helped resolve a long-standing conundrum in the field, i.e., how proteins with anticipated similar structures acquired the ability to transport substantially bulkier substrates than cations (Lyons et al., 2020). The first structures of a P4-ATPase were those of the yeast Drs2/Cdc50 transporter (Bai et al., 2019; Timcenko et al., 2019, 2021), followed by the structures of human ATP8A1/CDC50A and ATP11C/CDC50A (Hiraizumi et al., 2019; Nakanishi et al., 2020a,b). These structures reveal that PS is precisely recognized and coordinated via its headgroup in the core of the membrane (Figure 3). However, it is still unclear how the lipid is then released to the cytosolic leaflet. More structural and biochemical studies are needed to address this issue. Noteworthy, the biochemical characterization of P4-ATPases suggests a significantly lower lipid transfer rate compared to scramblases – between 2 to 25 lipids per second for flippases (Theorin et al., 2019), as compared to ≈ 10,000 lipids per second for scramblases. This difference is likely due to the large conformational changes required for flippases to catalyze lipid transport through an alternating-access mechanism, compared to the facilitated diffusion performed by scramblases (Goren et al., 2014).

In cells, P4-ATPase activity is regulated by interacting proteins, such as kinases and small GTP-binding proteins, as well as by phosphoinositides. In yeast, the *trans*-Golgi Drs2/Cdc50 flippase is autoinhibited by its N- and C-termini (Zhou et al., 2013; Azouaoui et al., 2017; Bai et al., 2019; Timcenko et al., 2019), which contain binding sites for the Arf-like protein Arl1 and the guanine nucleotide exchange factor Gea2, respectively (Chantalat et al., 2004; Tsai et al., 2013). The flippase activity is also activated by PI(4)P (Natarajan et al., 2009; Timcenko et al., 2019). Although association of Drs2 with Arl1 and Gea2 is essential for stimulation of Drs2 activity in native TGN membranes (Tsai et al., 2013), this requirement has not been recapitulated *in vitro*. In addition, the PM Dnf1/2 and Lem3 flippases as well as the human flippase ATP8A2 are positively regulated by kinases (Roelants et al., 2010; Chalat et al., 2017; Frøsig et al., 2020). During apoptosis, PS internalization is also abolished by caspase-mediated proteolytic cleavage of ATP11C concurrently with Xkr8 activation (Segawa et al., 2014).

## Lateral Organization of PS in Membranes

Segregation of lipids in the plane of the membrane has been established for the PM, with nanodomains made of cholesterol and sphingolipids being a prominent example (Levental et al., 2020). Several lines of evidence also point to lateral segregation of PS in the cytoplasmic leaflet of the PM. With the aid of electron microscopy techniques and genetically-encoded lipid biosensors, PS appears to cluster within domains of ∼11 nm in diameter in the inner leaflet of the PM of baby hamster kidney (BHK) cells (Fairn et al., 2011b). In a landmark study, Fairn et al. (2011a) observed accumulation of PS in the bud neck of *S. cerevisiae*, which was required for proper localization of the small GTP binding protein Cdc42 and the establishment of cell polarity. The uneven distribution of PS in the plane of the membrane is also highlighted by the accumulation of PS in caveolae (Fairn et al., 2011b). The relationship between PS and caveolae is two-sided: on the one hand, caveolae control PS clustering as knock-down of caveolin-1, the main structural component of caveolae, significantly increases PS clustering at the PM of BHK cells (Ariotti et al., 2014); on the other hand, both PS sequestration using a tandem Lact-C2 domain or stimulation of PS scrambling depletes caveolae. Such effect is specific to PS because selective depletion of PI(4,5)P_2_ or PI(4)P using a rapamycin inducible pseudojanin construct did not markedly induce disassembly of caveolae at the PM (Hirama et al., 2017a).

Further nanocluster organization of PS was suggested in the cytosolic leaflet of the PM of red blood cells and human skin fibroblasts using FRL electron microscopy in combination with anti-PS/PI antibodies and gold-conjugated secondary antibodies (Murate et al., 2015). Moreover, a recent study aiming at deciphering the selective interaction of the Influenza A virus matrix protein 1 (M1) with PS suggests that PS can laterally segregate in model membranes and at the PM of HEK293T cells, irrespective of the presence of M1 (Bobone et al., 2017).

Decreasing the cholesterol content of the PM using methyl-β-cyclodextrin drives massive relocalization of PS to endocytic membranes as a consequence of increased membrane curvature, a phenomenon that is exemplified by the recruitment of N-BAR domain-containing endophilin (which displays avidity for highly curved membranes) and synaptojanin (Daumke et al., 2014). Removal of cholesterol increases the lateral concentration of PS. Consequently, the surface charge density increases, thereby facilitating membrane bending for endocytosis (Hirama et al., 2017b). Repulsion of PS headgroups due to their negative charge can be overcome by cholesterol, which is suggested to act as a “spacer” between PS molecules.

The fact that decreasing the PS content disrupts proper transbilayer localization of cholesterol suggests that PS and cholesterol interact in the inner leaflet of the PM. This interaction highly depends on the exact nature of PS acyl chains (Maekawa and Fairn, 2015). Moreover, PS has been found to be an essential component of PM nanoclusters that are made of K-Ras and its downstream effectors. PS but not PI(4,5)P_2_ extensively co-localizes with K-Ras nanoclusters (Zhou et al., 2014), suggesting that K-Ras selectively interacts with PS. Noteworthy, cholesterol depletion does not alter nanoclustering of K-Ras in mammalian cells (Prior et al., 2003). Furthermore, restoration of K-Ras nanoclustering occurs once PS-depleted cells are supplemented with asymmetric PS species, i.e., PS with one saturated and one unsaturated acyl chain (16:0/18:1 and 18:0/18:1), but not by fully saturated PS species (Zhou et al., 2017).

In yeast, Nishimura et al. observed a synergistic effect of unsaturated PS and PI(4)P on the activity of the PI(4)P kinase PIP5K at the PM, which depended on the presence of Osh proteins. Using FRET, they observed a co-distribution of PS and PI(4)P in liposomes, which was enhanced by the presence of sterols. They suggested that Osh proteins promote the formation of nanodomains at the PM, required for optimal activity of PIP5K (Nishimura et al., 2019).

Molecular dynamic simulations using asymmetric membranes mimicking exosomes derived from PC-3 cells indicated interleaflet coupling between very long chain sphingomyelin species (C24) and lipids of the inner leaflet, with a preferred interdigitation occurring between SM 18:1/24:0 in the outer leaflet and PS 18:0/18:1 in the inner leaflet (Róg et al., 2016), which is the prevalent PS species in several mammalian cell types (Skotland and Sandvig, 2019). Thus, interdigitation may be an additional mean to regulate nanoscale organization of lipids (e.g., PS) in the cytosolic leaflet of membranes. Another example of PS lateral segregation was observed in yeast after selective extraction of the PM proteins Pma1 or Can1, which localize to different membrane domains. Using styrene maleic-acid lipid particles (SMALPs) for protein extraction and purification, the lipids co-purified with each protein, termed the periprotein lipidomes, were identified by mass spectrometry. The lipid fraction in the vicinity of Pma1 and Can1 was found enriched in PS and depleted in ergosterol compared to the overall membrane. Such enrichment in PS and depletion in ergosterol would provide an adequate environment for large conformational changes of membrane proteins to take place in the otherwise highly ordered yeast PM (van ’t Klooster et al., 2020).

Within internal organelles, the most striking example of lateral lipid segregation has been observed in the yeast vacuoles in response to starvation or stress conditions (Toulmay and Prinz, 2013). The formation of these large (micron-scale) stable domains appears lipid-driven and is sterol-dependent. The distribution of PS in this system has not been addressed. However, another very recently published report describes formation of stable lipid domains at the PM of yeast cells that lack PS (*cho1Δ*) and are grown at an elevated temperature under non-starvation conditions (Mioka et al., 2021). These domains, termed “void zones,” are devoid of proteins and also of many phospholipid species, and it is proposed that they represent sterol and sphingolipid-rich domains that form when PS is not available to promote lipid mixing. The formation of void zones also requires transbilayer asymmetry because it is abolished when PM flippases are deleted. These data underline the importance of PS for PM organization and function.

In the ER, the existence of PS-enriched domains was postulated based on the observation that PS-synthetase activity was enriched in biochemically-purified MAM (Vance, 1990; Gaigg et al., 1995). However, lateral inhomogeneity in the distribution of PS within the ER has not been directly observed, which is not surprising, given the difficulties in imaging of internal membranes and the size and dynamics of the PS nanodomains observed at the PM. Tsuji et al. (2019) observed a higher concentration of PS in the nuclear compared to the ER membrane, which, given the continuity of this membrane system, would require a diffusion barrier that retains more PS in the nuclear membrane. Several studies have reported the existence of diffusion barriers in the ER, which could affect the flow of lipids. A sphingolipid-based diffusion barrier has been proposed to exist in the nuclear membrane and in the cortical ER at the bud-neck region of yeast (Clay et al., 2014). A recent study measuring bilayer thickness of the ER revealed thickening of the membrane in the plane of cleavage between mother and daughter cell, as well as at ER-trans-Golgi contact sites (Prasad et al., 2020). This thickening depended on long-chain ceramides rather than sphingolipids and acted as a diffusion barrier for transmembrane proteins.

## Phosphatidylserine Transport During Autophagy

Recent studies of the autophagy pathway highlight the importance of PS for this process and can be used as an example for how different transport mechanisms can contribute to the spatial organization of this lipid. During autophagy, a large amount of phospholipid is required for the formation of isolation membranes, precursors of autophagosomes. A long-standing question in the field has been the source of this membrane. It was presumed that the autophagosomal membrane is derived from other pre-existing membranes that are delivered via vesicular trafficking. The composition of the autophagosomal membrane and the proximity of autophagosomes to the ER, particularly to the ER exit sites from which the COPII transport vesicles bud, pointed to an important contribution of the COPII transport pathway delivering ER membrane to the growing autophagosome (Jensen and Schekman, 2011; Shima et al., 2019). However, recent work suggests that transfer of lipids *via* LTPs, and even *de novo* lipid synthesis, make an important contribution to the delivery of lipids required for growth of the isolation membrane.

One of the proteins required for autophagosome formation is Atg2. Interestingly, stretches of primary sequence similarity have been noted between Atg2 and Vps13 (Velikkakath et al., 2012). Similarities occur in the first ∼120 residues at the N terminus (referred to as the Chorein_N domain) and in a stretch of ∼70 residues in the C-terminal region (termed ATG_C in Pfam). These similarities and the reconstitution of Atg2 activity on synthetic membranes suggest that, like Vps13, Atg2 is an LTP acting as a bridge to mediate fast flow of phospholipids from the ER to the isolation membrane (Maeda et al., 2019; Osawa et al., 2019; Valverde et al., 2019). To provide directionality to this flow, Atg2 activity may be coupled with phospholipid synthesis at the ER (Schütter et al., 2020). On the other end, lipid scramblase activity of the autophagosomal protein Atg9 could provide a sink for the phospholipids delivered by Atg2 (Ghanbarpour et al., 2021). This is supported by the observation that purified Atg9 facilitates transbilayer transport of NBD-PS, -PC and -PE, as well as natural PI(3)P (Maeda et al., 2020; Matoba et al., 2020). It remains to be determined whether PI(4)P is also a transport substrate of Atg9. Ghanbarpour et al. (2021) further show that ATG2 interacts with TMEM41B/VMP1, an ER protein complex that displays scramblase activity toward NBD-PC, -PS and -PE (Li et al., 2020). Reequilibration of phospholipids by TMEM41B/VMP1 and ATG9 as they are extracted from the ER and delivered to the isolation membrane, respectively, would promote proper stability and expansion of membranes, and ensure efficient lipid shuttling. In agreement with the scramblase activity of Atg9, PS, PI(4)P, and PC were found evenly distributed over the two leaflets of autophagosomes and autophagic bodies, in contrast with a predominantly cytosolic localization of PS at the vacuole (Orii et al., 2021). An impressive reconstitution of the activities of Atg9, Atg2, and a large (near full) number of other Atg proteins in artificial membranes paves the way to a detailed understanding of these processes (Sawa-Makarska et al., 2020).

## Conclusion and Perspectives

Our understanding of PS distribution within cell membranes has greatly improved in recent years, benefiting from the combination of cutting-edge cell biological, biochemical and biophysical techniques. For example, such an interdisciplinary approach revealed that lipid acyl chain unsaturation, including PS, varies between membrane leaflets, and that this acyl chain asymmetry is tightly linked with lipid packing and protein transmembrane domain asymmetry in individual leaflets (Lorent et al., 2020). We expect that advances in lipidomics and lipidomic imaging will help provide a comprehensive map of PS and lipid acyl chain distribution in cell membranes in the next few years, which can be related to its functional relevance, as was for example recently demonstrated by Shindou et al. (2017). Whereas much progress has been made toward understanding of the organization of PS at the PM, the spatial distribution of PS in internal membranes remains enigmatic. This is hardly surprising, given the difficulties in tracking of phospholipids inside cells at the required spatial and temporal resolution and the complexity and plasticity of lipid transport pathways. However, the field is developing rapidly thanks to an increased interest in the cell biology of lipids, ingenuity and technical advance. On one hand, recent years have brought intense developments in high/super-resolution imaging of cellular membranes, in specific fluorescent probes and reporters of membrane polarity or fluidity/disorder (Sot et al., 2021) and in novel electron microscopy techniques (Heberle et al., 2020). On the other hand, *in vitro* reconstitution and structural studies have greatly improved our understanding of LTP function at MCS and of elementary but previously elusive PS movements (Timcenko et al., 2019; de la Mora et al., 2021). The reconstitution of the autophagy pathway is a spectacular example of a complex reconstituted reaction showing coupling between different lipid transport proteins (Sawa-Makarska et al., 2020). Undoubtedly, many more such successful *in vitro* reconstitution of cellular processes is yet to come.

## Author Contributions

GL, JD’A, TD, and AČ conceptualized and wrote the manuscript. All authors contributed to the article and approved the submitted version.

## Conflict of Interest

The authors declare that the research was conducted in the absence of any commercial or financial relationships that could be construed as a potential conflict of interest.

## Publisher’s Note

All claims expressed in this article are solely those of the authors and do not necessarily represent those of their affiliated organizations, or those of the publisher, the editors and the reviewers. Any product that may be evaluated in this article, or claim that may be made by its manufacturer, is not guaranteed or endorsed by the publisher.
